# Supplementary material for: Histone variant H3.3 residue S31 is essential for Xenopus gastrulation regardless of the deposition pathway
Source: Nat Commun. 2020 Mar 9;11:1256. doi: 10.1038/s41467-020-15084-4 (PMC7062693; doi:10.1038/s41467-020-15084-4)
Supplement: Supplementary file 3 — Description of Additional Supplementary Files [file 41467_2020_15084_MOESM3_ESM.pdf]

Description of Additional Supplementary Files

File Name: Supplementary Movie 1
Description: Endogenous H3.3 depletion leads to gastrulation defects. Left embryo is injected with eH3.3 WT mRNA while the middle embryo is injected with 4.6ng of morpholino against H3.3. Right embryo is injected with both morpholino and eH3.3 WT mRNA.

File Name: Supplementary Movie 2
Description: Dose-dependent H3.3 depletion defects. From left to right, the first three sets of embryos have been injected with 4.6ng, 9.2ng, 18.4ng of morpholino against H3.3, respectively. Right embryos have not been injected. Two replicates for each condition are shown.

File Name: Supplementary Data 1
Description: Antibody list

File Name: Supplementary Data 2
Description: Mass spectrometry data
