# Supplementary material for: Histone variant H3.3 residue S31 is essential for Xenopus gastrulation regardless of the deposition pathway
Source: Nat Commun. 2020 Mar 9;11:1256. doi: 10.1038/s41467-020-15084-4 (PMC7062693; doi:10.1038/s41467-020-15084-4)
Supplement: Supplementary file 6 — Supplementary Data 1 [file 41467_2020_15084_MOESM6_ESM.pdf]

Target	Source of antibody	Previous use	WB/IF here
HA	#11867423001, clone 3F10, Sigma	(Szenker et al. 2012)	WB:1/1000
H3S10ph	Mabi0312, Wako	(Kimura et al. 2008)	WB: 1/1000; IF: 1/1000
H3.3S31ph	ab92628, lot GR147052-3, Abcam	(Martire et al. 2019)	WB: 1/1000; IF: 1/200
	39637, lot 23009001, Active Motif	(Armache et al. 2019)	WB: 1/1000
H3.3	M01, Abnova	(Szenker et al. 2012)	WB: 1/200
	#09-838, lot 3085003, Millipore	(Martire et al. 2019)	WB: 1/500
H4	ab31830, Abcam	(Boyarchuk et al. 2014)	WB: 1/1000
α-Tubulin	T9026, Sigma	(Szenker et al. 2012)	WB: 1/5000
H3	ab1791, Abcam	(Lacoste et al. 2014)	WB: 1/5000
H3 (N-ter)	H9289, Sigma	(Lacoste et al. 2014)	WB: 1/1000
xEmi2	Lab-made	Gift from Mary Dasso	WB: 1/1000
xHIRA	Lab-made	(Ray-Gallet et al. 2002)	WB: 1/1000
xDAXX	Lab-made	Gift from Mary Dasso	WB: 1/500
xp60	Lab-made, Agro Bio	(Ray-Gallet et al. 2007)	WB: 1/1000
xCENP-A	Lab-made	Gift from Aaron Straight)	WB: 1/500
xp150	Lab-made, Agro Bio	(Quivy et al. 2001)	WB: 1/1000
H3K27ac	ab4729, lot GR 3231937_1, Abcam	/	WB: 1/1000
	39133, lot 28518012, Active Motif	(Martire et al. 2019)	WB: 1/1000
H3K27me3	39155, lot 6517018, Active Motif	/	WB: 1/1000
H3K36me3	ab9050, lot GR 3265404-1, Abcam	histone antibodies.com	WB: 1/1000
	61021, clone 0333, Active Motif	(Armache et al. 2019)	WB: 1/1000
hCENP-A	#2186, Cell Signaling	(Lacoste et al. 2014)	WB: 1/500
hHIRA	39557, clone WC 119.2H11, Active Motif	(Ray-Gallet et al. 2011)	WB: 1/200
hDAXX	#4533, clone 25C12, Cell Signaling	(Lacoste et al. 2014)	WB: 1/1000
hHJURP	HPA008436, lot A33704, Sigma	(Lacoste et al. 2014)	WB: 1/1000
hp60	Lab-made, Agro Bio	(Ray-Gallet et al. 2011)	WB: 1/1000
H3S28ph	#07-145, lot		

	3079709, Millipore	(Martire et al. 2019)	WB: 1/1000
	ab32388, clone E191, lot GR 65021_3, Abcam	(Armache et al. 2019	WB: 1/1000
H4K8ac	#07-328, Millipore	histone antibodies.com	WB: 1/1000
H3T3ph	#07-424, lot 3012075, Millipore	/	WB: 1/1000
H3K4me1	#305-34799, clone MABI0302, Wako	(Kimura et al. 2008)	WB: 1/1000

References

Armache A, Yang S, Robbins LE, Durmaz C, Daman CD, Jeong JQ, Martinez de Paz A, Ravishankar A, Arslan T, Lin S et al. 2019. Phosphorylation of the ancestral histone variant H3.3 amplifies stimulation-induced transcription. *bioRxiv*.

Boyarchuk E, Filipescu D, Vassias I, Cantaloube S, Almouzni G. 2014. Pericentric heterochromatin state during the cell cycle controls the histone variant composition of centromeres. *J Cell Sci* **127**: 3347-3359.

Kimura H, Hayashi-Takanaka Y, Goto Y, Takizawa N, Nozaki N. 2008. The organization of histone H3 modifications as revealed by a panel of specific monoclonal antibodies. *Cell Struct Funct* **33**: 61-73.

Lacoste N, Woolfe A, Tachiwana H, Villar Garea A, Barth T, Cantaloube S, Kurumizaka H, Imhof A, Almouzni G. 2014. Mislocalization of the Centromeric Histone Variant CenH3/CENP-A in Human Cells Depends on the Chaperone DAXX. *Mol Cell* **53**: 631-644.

Martire S, Gogate AA, Whitmill A, Tafessu A, Nguyen J, Teng YC, Tastemel M, Banaszynski LA. 2019. Phosphorylation of histone H3.3 at serine 31 promotes p300 activity and enhancer acetylation. *Nat Genet* **51**: 941-946.

Quivy JP, Grandi P, Almouzni G. 2001. Dimerization of the largest subunit of chromatin assembly factor 1- importance in vitro and during Xenopus early development. *Embo J* **20**: 2015-2027.

Ray-Gallet D, Quivy JP, Scamps C, Martini EM, Lipinski M, Almouzni G. 2002. HIRA Is Critical for a Nucleosome Assembly Pathway Independent of DNA Synthesis. *Mol Cell* **9**: 1091-1100.

Ray-Gallet D, Quivy JP, Sillje HW, Nigg EA, Almouzni G. 2007. The histone chaperone Asf1 is dispensable for direct de novo histone deposition in Xenopus egg extracts. *Chromosoma* **116**: 487-496.

Ray-Gallet D, Woolfe A, Vassias I, Pellentz C, Lacoste N, Puri A, Schultz DC, Pchelintsev NA, Adams PD, Jansen LE et al. 2011. Dynamics of histone H3 deposition in vivo reveal a nucleosome gap-filling mechanism for H3.3 to maintain chromatin integrity. *Mol Cell* **44**: 928-941.

Szenker E, Lacoste N, Almouzni G. 2012. A developmental requirement for HIRA-dependent H3.3 deposition revealed at gastrulation in Xenopus. *Cell Rep* **1**: 730-740.
