# Supplementary material for: Histone variant H3.3 residue S31 is essential for Xenopus gastrulation regardless of the deposition pathway
Source: Nat Commun. 2020 Mar 9;11:1256. doi: 10.1038/s41467-020-15084-4 (PMC7062693; doi:10.1038/s41467-020-15084-4)
Supplement: Supplementary file 8 — Reporting Summary [file 41467_2020_15084_MOESM8_ESM.pdf]

nature research

Corresponding author(s): Geneviève Almouzni

Last updated by author(s): Jan 25, 2020

Reporting Summary

Nature Research wishes to improve the reproducibility of the work that we publish. This form provides structure for consistency and transparency in reporting. For further information on Nature Research policies, see Authors & Referees and the Editorial Policy Checklist.

Statistics

For all statistical analyses, confirm that the following items are present in the figure legend, table legend, main text, or Methods section.

n/a	Confirmed	
☐	☒	The exact sample size (*n*) for each experimental group/condition, given as a discrete number and unit of measurement
☐	☒	A statement on whether measurements were taken from distinct samples or whether the same sample was measured repeatedly
☒	☐	The statistical test(s) used AND whether they are one- or two-sided<br>*Only common tests should be described solely by name; describe more complex techniques in the Methods section.*
☒	☐	A description of all covariates tested
☒	☐	A description of any assumptions or corrections, such as tests of normality and adjustment for multiple comparisons
☒	☐	A full description of the statistical parameters including central tendency (e.g. means) or other basic estimates (e.g. regression coefficient) AND variation (e.g. standard deviation) or associated estimates of uncertainty (e.g. confidence intervals)
☒	☐	For null hypothesis testing, the test statistic (e.g. *F*, *t*, *r*) with confidence intervals, effect sizes, degrees of freedom and *P* value noted<br>*Give P values as exact values whenever suitable.*
☒	☐	For Bayesian analysis, information on the choice of priors and Markov chain Monte Carlo settings
☒	☐	For hierarchical and complex designs, identification of the appropriate level for tests and full reporting of outcomes
☒	☐	Estimates of effect sizes (e.g. Cohen's *d*, Pearson's *r*), indicating how they were calculated

Our web collection on statistics for biologists contains articles on many of the points above.

Software and code

Policy information about availability of computer code

Data collection	No software was used in this study.
Data analysis	No software was used in this study.

For manuscripts utilizing custom algorithms or software that are central to the research but not yet described in published literature, software must be made available to editors/reviewers. We strongly encourage code deposition in a community repository (e.g. GitHub). See the Nature Research guidelines for submitting code & software for further information.

Data

Policy information about availability of data

All manuscripts must include a data availability statement. This statement should provide the following information, where applicable:

- Accession codes, unique identifiers, or web links for publicly available datasets
- A list of figures that have associated raw data
- A description of any restrictions on data availability

Raw data for Mass Spectrometry are available in Pride (PXD016497)

Field-specific reporting

Please select the one below that is the best fit for your research. If you are not sure, read the appropriate sections before making your selection.

☒ Life sciences ☐ Behavioural & social sciences ☐ Ecological, evolutionary & environmental sciences

For a reference copy of the document with all sections, see nature.com/documents/nr-reporting-summary-flat.pdf

Life sciences study design

All studies must disclose on these points even when the disclosure is negative.

Sample size	Sample size was defined according to the maximum possible amount of embryos to inject in the 30-40min window time frame at the 2-cell stage of Xenopus laevis development.
Data exclusions	No data was excluded in this study.
Replication	All experiments have been replicated at least 3 times in this study.
Randomization	No groups were needed for this study.
Blinding	Blinding was not performed in this study since the output is whether embryos close or not their blastopore.

Reporting for specific materials, systems and methods

We require information from authors about some types of materials, experimental systems and methods used in many studies. Here, indicate whether each material, system or method listed is relevant to your study. If you are not sure if a list item applies to your research, read the appropriate section before selecting a response.

Materials & experimental systems

n/a	Involved in the study	
☐	☒	Antibodies
☐	☒	Eukaryotic cell lines
☒	☐	Palaeontology
☐	☒	Animals and other organisms
☒	☐	Human research participants
☒	☐	Clinical data

Methods

n/a	Involved in the study	
☒	☐	ChIP-seq
☒	☐	Flow cytometry
☒	☐	MRI-based neuroimaging

Antibodies

Antibodies used

Find below the exact list of antibodies used in this study (also available in Table 1).
HA (3F10, Roche)
H3.3S10ph (Mabi0312, Wako)
H3.3S31ph (ab92628, Abcam)
H3.3S31ph 39637, Active Motif)
H3.3 (M01, Abnova)
H3.3 (09-838, Millipore)
H4 (05-858, Upstate)
α-Tubulin (T9026, Sigma)
H3 (ab1791, Abcam)
H3 N-ter (H9289, Sigma)
xEmi2 (From Mary Dasso)
xHIRA (Agro Bio)
xDAXX (From Mary Dasso)
xp60 (Agro Bio)
xCENP-A (From Aaron Straight)
xp150 (Agro Bio)
H3K27ac (ab4729, Abcam)
H3K27ac (39133, Active Motif)
H3K27me3 (39155, Active Motif)
H3K36me3 (ab9050, Abcam)
H3K36me3 (61021, Active Motif)
hCENP-A (2186, Cell Signaling)
hHIRA (39557, Active Motif)
hDAXX (4533, Cell Signaling)
hHJURP (HPA008436, Sigma)
hp60 (Agro Bio)
H3S28ph (07-145, Millipore)
H3S28ph (ab32388, Abcam)
H4k8ac (07-328, Millipore)
H3T3ph (07-424, Millipore)
H3K4me1 (305-34799, Wako)

Validation

Most of our antibodies have been already characterized in previous publications from our group or others, and we have further

tested antibodies for H3 PTMs with unmodified H3 peptides, which invalidated H3K27ac from Active Motif (see Supplemental figure 8).

Eukaryotic cell lines

Policy information about cell lines

Cell line source(s)	HeLa B (Homo sapiens, ATCC), Flp-In T-Rex 293 (Homo sapiens, Invitrogen), and A6 (Xenopus laevis, ATCC).
Authentication	None of the cells used were authenticated in this study.
Mycoplasma contamination	HeLa B, Flp-In T-Rex 293, and A6 cell lines were tested negative for mycoplasma contamination.
Commonly misidentified lines (See ICLAC register)	This study did not involve misidentified cell lines.

Animals and other organisms

Policy information about studies involving animals; ARRIVE guidelines recommended for reporting animal research

Laboratory animals	As stated in the material and methods section of the manuscript, Xenopus laevis (females for eggs collection and males for sperm collection) from the Centre de Ressource Biologie Xenope of Rennes were used in this study. All adults were more than 2 years old.
Wild animals	This study did not involve wild animals captured in a field.
Field-collected samples	This study did not involve samples collected in the field.
Ethics oversight	As stated in the material and methods section of the manuscript, animal care and use for this study were performed in accordance with the recommendations of the European Community (2010/63/UE) for the care and use of laboratory animals. Experimental procedures were specifically approved by the ethics committee of the Institut Curie CEEA-IC #118 (Authorization APAFIS#11226-3992017091116031353-v1 given by National Authority) in compliance with the international guidelines. David Sitbon, Ekaterina Boyarchuk and Geneviève Almouzni possess the Authorization for vertebrates' experimental use.

Note that full information on the approval of the study protocol must also be provided in the manuscript.
