# Supplementary material for: Histone variant H3.3 residue S31 is essential for Xenopus gastrulation regardless of the deposition pathway
Source: Nat Commun. 2020 Mar 9;11:1256. doi: 10.1038/s41467-020-15084-4 (PMC7062693; doi:10.1038/s41467-020-15084-4)
Supplement: Supplementary file 9 — Source Data [file 41467_2020_15084_MOESM9_ESM.pdf]

Figure 2 B

Conditions :	Control	MO H3.3	MO H3.3 + H3.2 WT	MO H3.3 + H3.3 WT	MO H3.3 + H3.3 A87S	MO H3.3 + H3.3 I89V	MO H3.3 + H3.3 G90M	MO H3.3 + H3.3 svm
Numbers of injected eggs :	30	36	33	36	33	30	36	32
Dead embryos:	1	32	31	9	10	8	13	11
% of proper developped eggs :	96,66667	11,11111111	6,060606061	75	69,6969697	73,33333333	63,88888889	65,625
Conditions :	Control	MO H3.3	MO H3.3 + H3.2 WT	MO H3.3 + H3.3 WT	MO H3.3 + H3.3 A87S	MO H3.3 + H3.3 I89V	MO H3.3 + H3.3 G90M	MO H3.3 + H3.3 svm
Numbers of injected eggs :	30	44	48	48	30	30	32	42
Dead embryos:	1	42	48	16	8	7	8	16
% of proper developped eggs :	96,66667	4,545454545	0	66,66666667	73,33333333	76,66666667	75	61,9047619
Conditions :	Control	MO H3.3	MO H3.3 + H3.2 WT	MO H3.3 + H3.3 WT	MO H3.3 + H3.3 A87S	MO H3.3 + H3.3 I89V	MO H3.3 + H3.3 G90M	MO H3.3 + H3.3 svm
Numbers of injected eggs :	30	35	32	30	31	34	30	33
Dead embryos:	0	32	31	9	11	9	10	12
% of proper developped eggs :	100	8,571428571	3,125	70	64,51612903	73,52941176	66,66666667	63,63636364
Average stage 12	97,77778	8,075998076	3,061868687	70,55555556	69,18214402	74,50980392	68,51851852	63,72204185
St dev	1,924501	3,31074766	3,030796204	4,194352464	4,431089824	1,870469022	5,782405554	1,861598356

Figure 3A

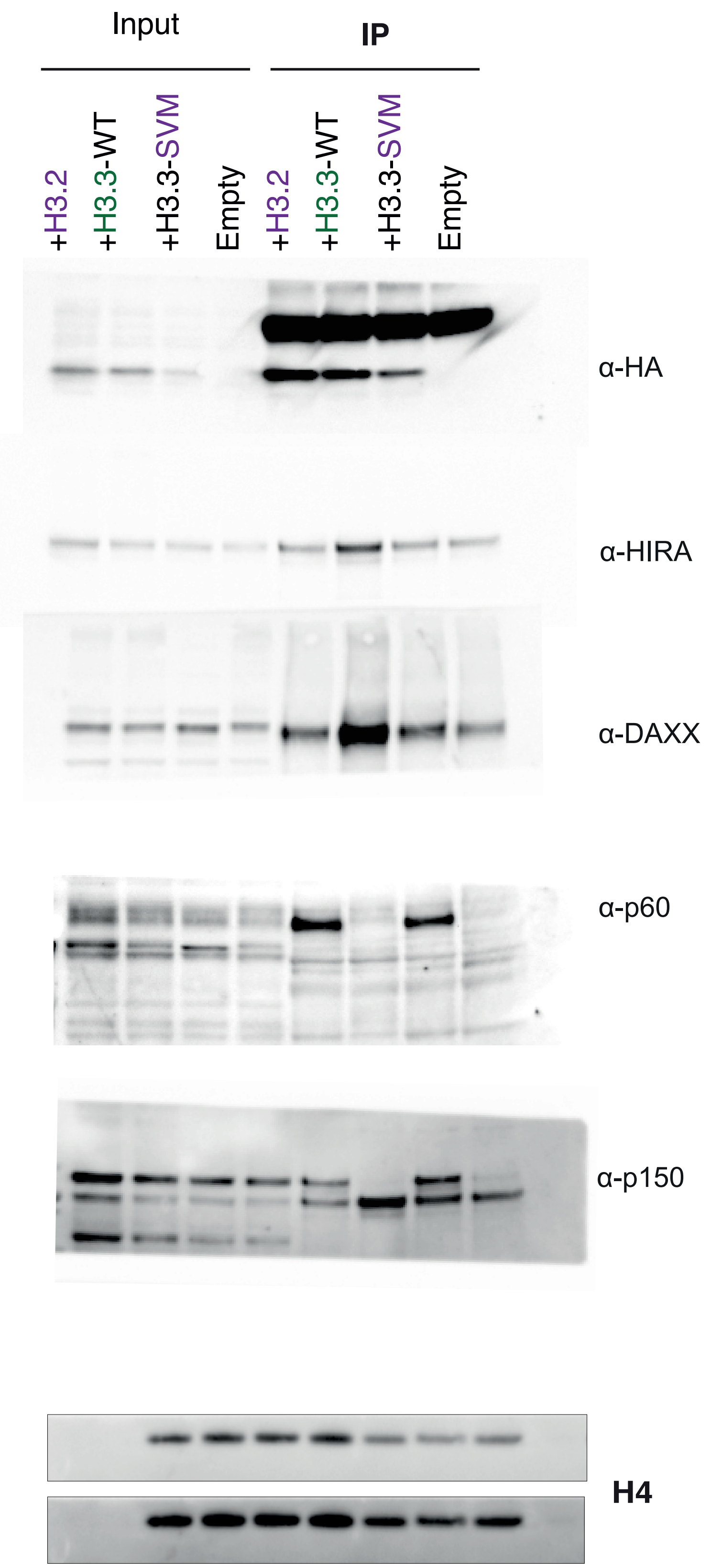
Input
IP
+H3.2
+H3.3-WT
+H3.3-SVM
Empty
+H3.2
+H3.3-WT
+H3.3-SVM
Empty
α-HA
α-HIRA
α-DAXX
α-p60
α-p150
H4

Figure 3B & Sup 3C

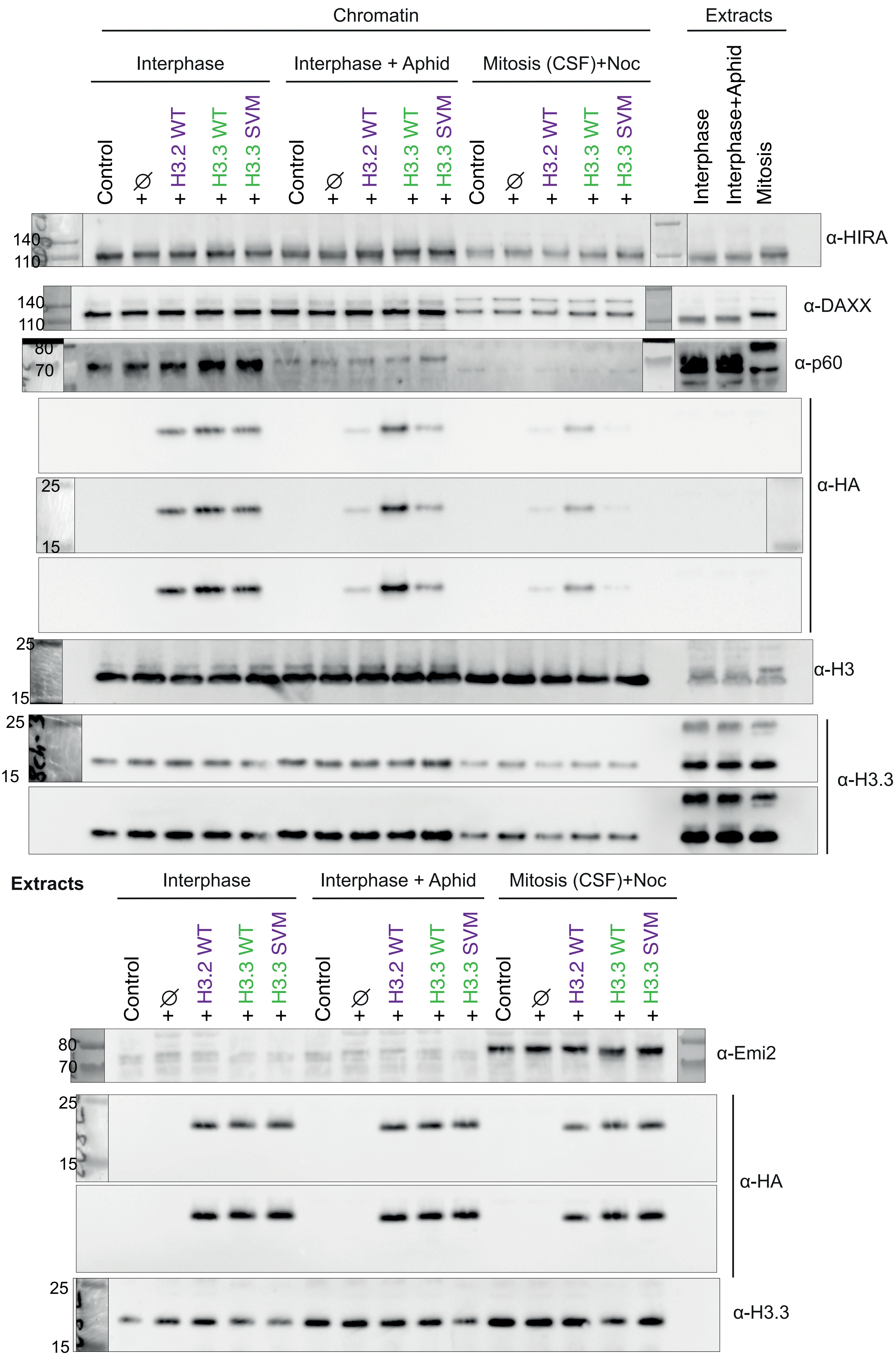

Figure 4 A

Conditions :	Control	MO H3.3 + H3.3 WT	MO H3.3 + H3.3 S31A
Numbers of injected eggs :	30	24	22
Dead embryos:	1	4	20
% of proper developped eggs :	96,66667	83,33333333	9,090909091
Conditions :	Control	MO H3.3 + H3.3 WT	MO H3.3 + H3.3 S31A
Numbers of injected eggs :	32	21	25
Dead embryos:	0	3	22
% of proper developped eggs :	100	85,71428571	12
Conditions :	Control	MO H3.3 + H3.3 WT	MO H3.3 + H3.3 S31A
Numbers of injected eggs :	30	27	23
Dead embryos:	2	5	21
% of proper developped eggs :	93,33333	81,48148148	8,695652174
Average stage 12	96,66667	83,50970018	9,928853755
St dev	3,333333	2,121906422	1,804519904

Figure 4C

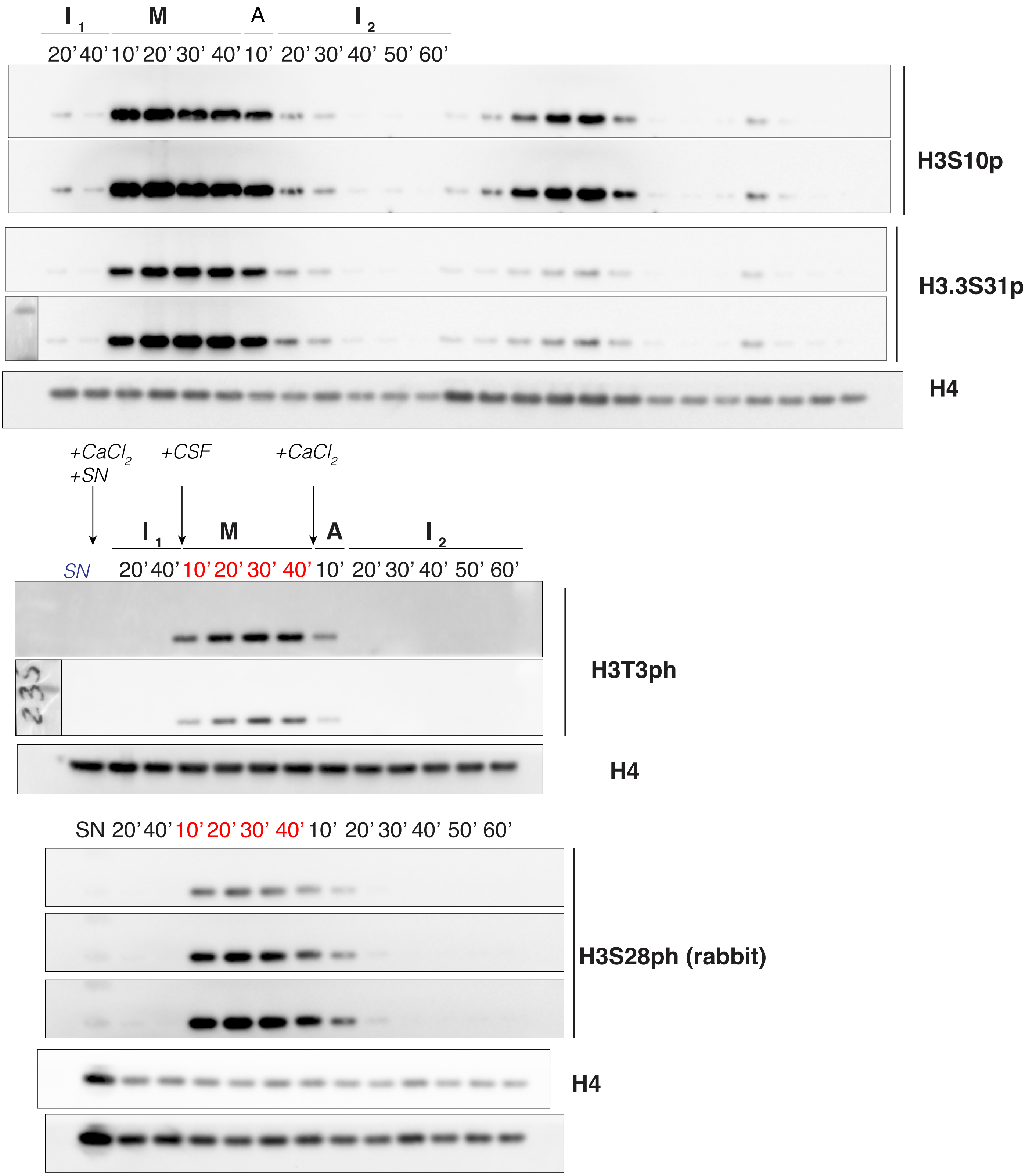
I 1
M
A
I 2
20' 40' 10' 20' 30' 40' 10' 20' 30' 40' 50' 60'
H3S10p
H3.3S31p
H4
+CaCl2
+SN
+CSF
+CaCl2
SN
20' 40' 10' 20' 30' 40' 10' 20' 30' 40' 50' 60'
H3T3ph
H4
SN 20' 40' 10' 20' 30' 40' 10' 20' 30' 40' 50' 60'
H3S28ph (rabbit)
H4

Figure 4D

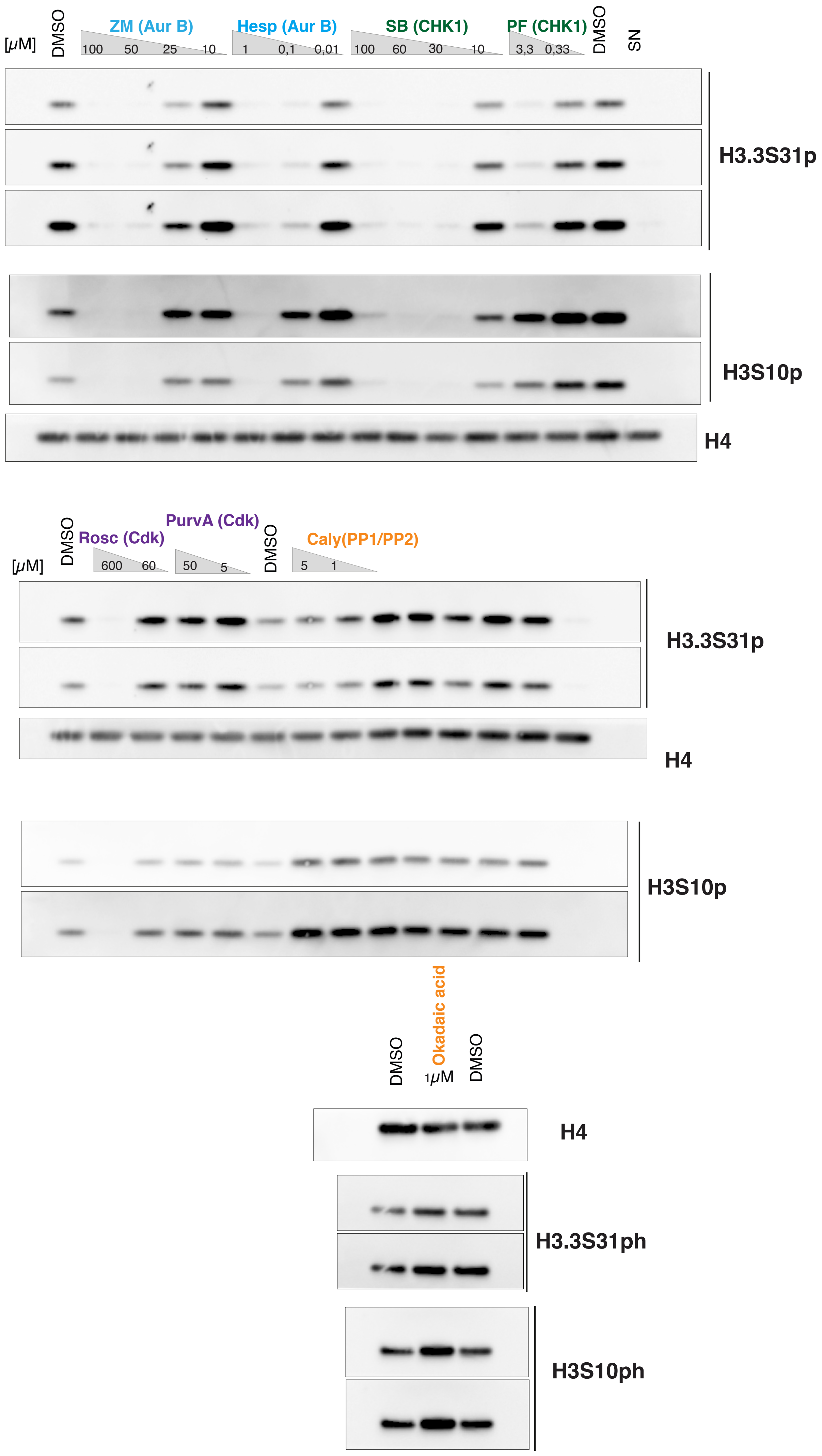
[μM]
DMSO
ZM (Aur B)
100 50 25 10
Hesp (Aur B)
1 0,1 0,01
SB (CHK1)
100 60 30 10
PF (CHK1)
3,3 0,33
DMSO
SN
H3.3S31p
H3S10p
H4
[μM]
DMSO
Rosc (Cdk)
600 60
PurvA (Cdk)
50 5
DMSO
Caly(PP1/PP2)
5 1
H3.3S31p
H4
H3S10p
DMSO
Okadaic acid
1μM
DMSO
H4
H3.3S31ph
H3S10ph

Figure 4D

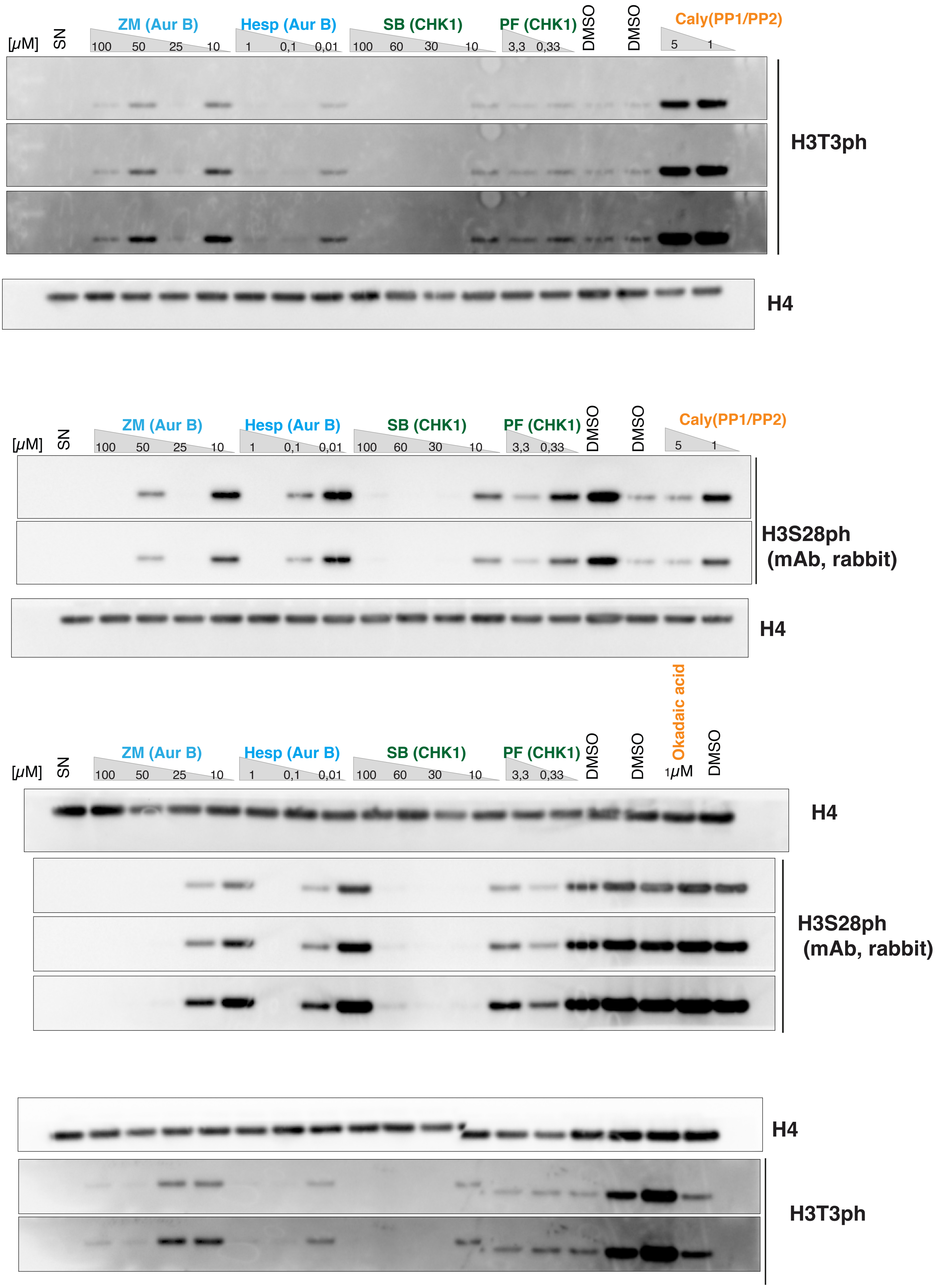

[μM]
SN
ZM (Aur B)
100 50 25 10
Hesp (Aur B)
1 0,1 0,01
SB (CHK1)
100 60 30 10
PF (CHK1)
3,3 0,33
DMSO
DMSO
Caly(PP1/PP2)
5 1
H3T3ph
H4
H3S28ph (mAb, rabbit)
Okadaic acid
1μM

Figure 5 A

Conditions :	Control	MO H3.3	MO H3.3 + H3.3 WT	MO H3.3 + H3.2 WT	MO H3.3 + H3.3 S31A	MO H3.3 + H3.3 S31D
Numbers of injected eggs :	20	35	40	39	42	40
Dead embryos:	0	31	10	36	34	14
% of proper developped eggs :	100	11,42857143	75	7,692307692	19,04761905	65

Conditions :	Control	MO H3.3	MO H3.3 + H3.3 WT	MO H3.3 + H3.2 WT	MO H3.3 + H3.3 S31A	MO H3.3 + H3.3 S31D
Numbers of injected eggs :	35	32	32	31	32	50
Dead embryos:	2	29	5	31	27	12
% of proper developped eggs :	94,28571	9,375	84,375	0	15,625	76

Conditions :	Control	MO H3.3	MO H3.3 + H3.3 WT	MO H3.3 + H3.2 WT	MO H3.3 + H3.3 S31A	MO H3.3 + H3.3 S31D
Numbers of injected eggs :	40	34	34	32	36	34
Dead embryos:	0	30	8	30	30	12
% of proper developped eggs :	100	11,76470588	76,47058824	6,25	16,66666667	64,70588235

Average stage 12	98,09524	10,85609244	78,61519608	4,647435897	17,11309524	68,56862745
St dev	3,299144	1,293627728	5,042039602	4,088894494	1,754438411	6,43743736

Figure 5B

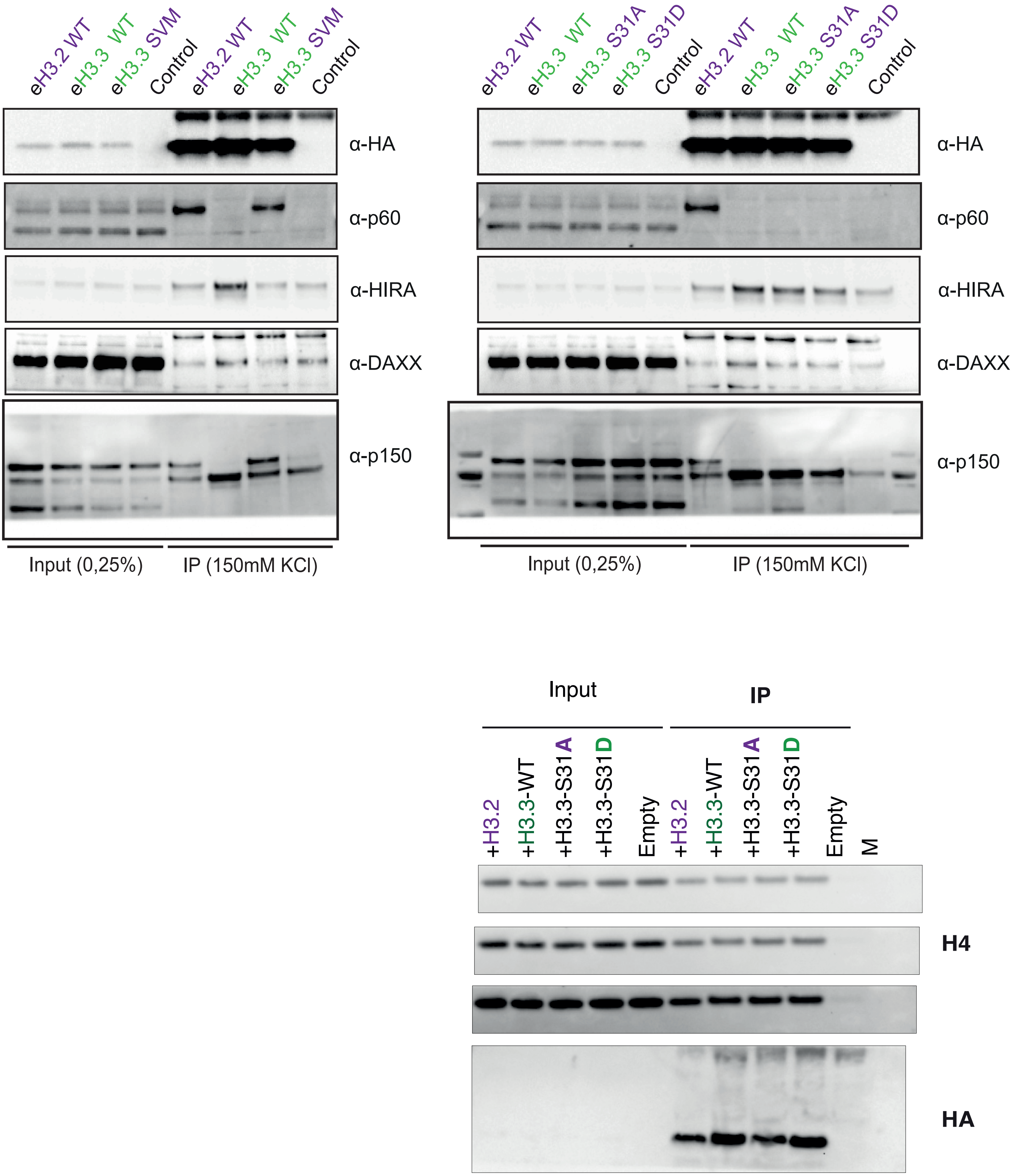
eH3.2 WT
eH3.3 WT
eH3.3 SVM
Control
eH3.3 S31A
eH3.3 S31D
α-HA
α-p60
α-HIRA
α-DAXX
α-p150
Input (0,25%)
IP (150mM KCl)
Input
IP
+H3.2
+H3.3-WT
+H3.3-S31A
+H3.3-S31D
Empty
M
H4
HA

Figure 5C and Sup 5C

Chromatin

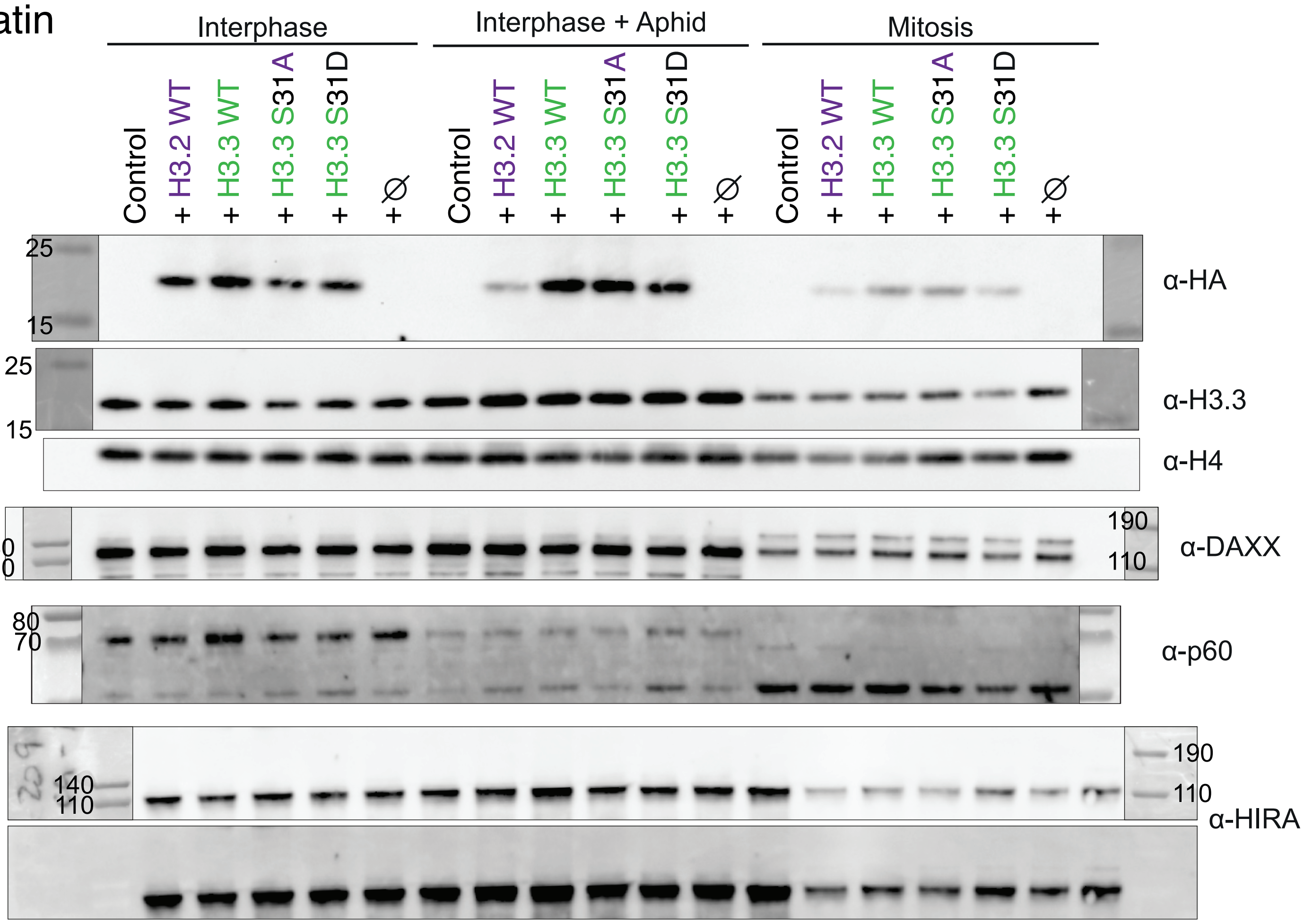

Extracts

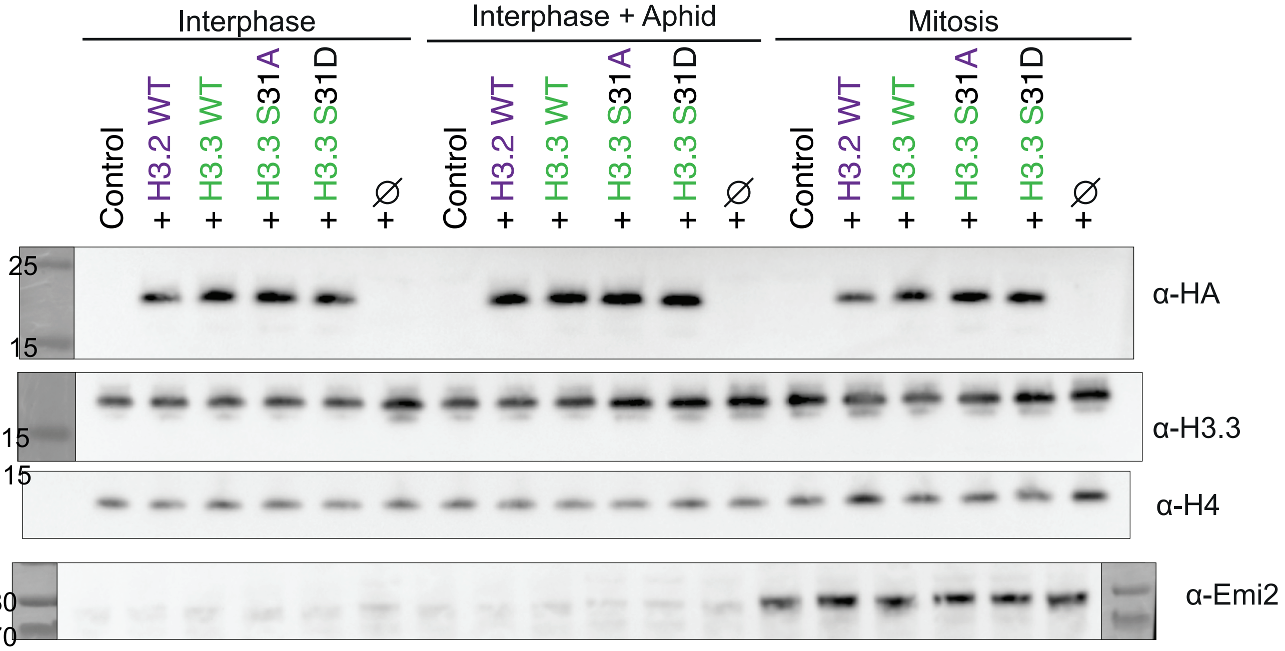

Figure 7B

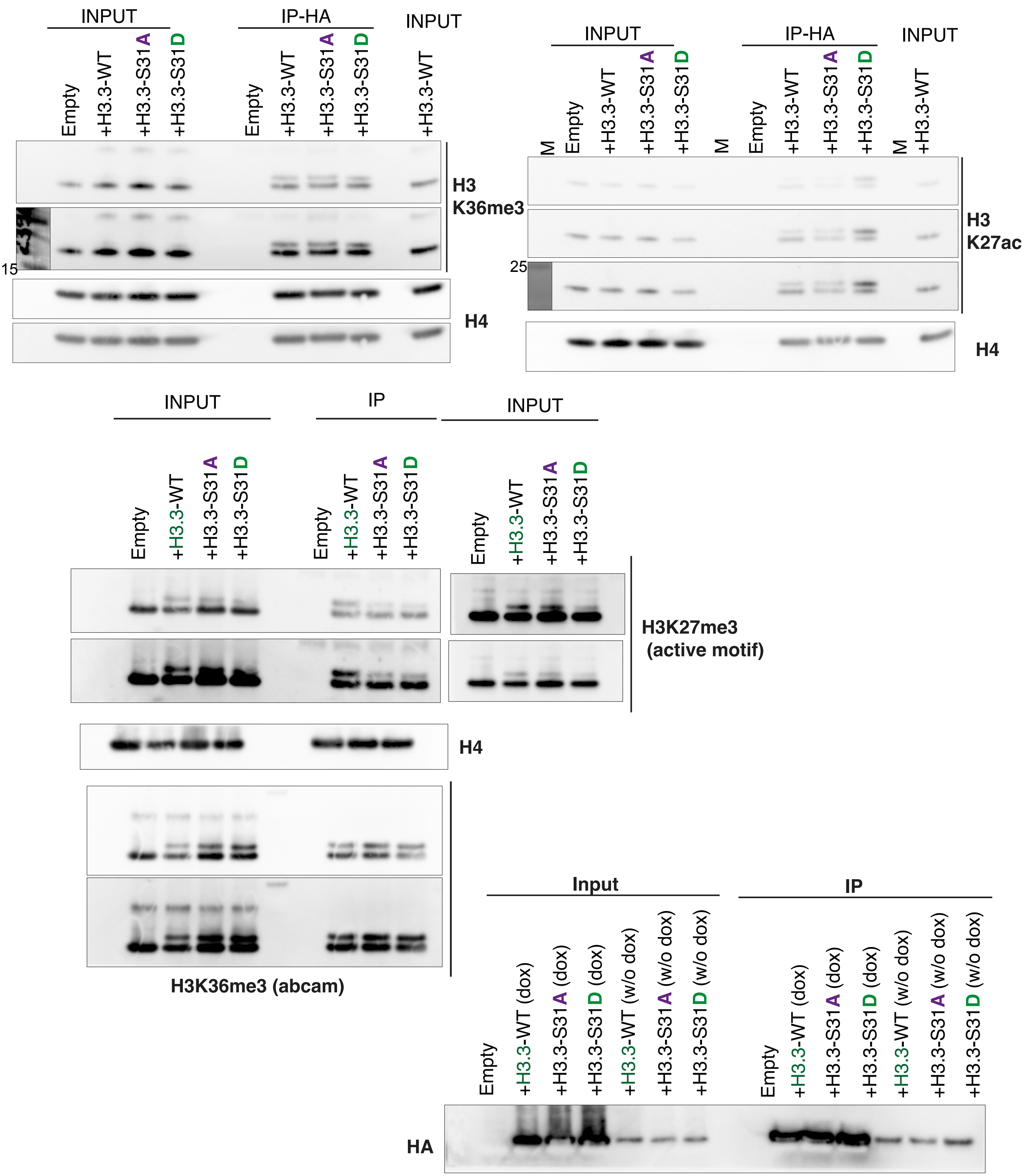
INPUT
IP-HA
INPUT
Empty
+H3.3-WT
+H3.3-S31A
+H3.3-S31D
M
H3 K36me3
H4
H3 K27ac
15
25
IP
H3K27me3 (active motif)
H3K36me3 (abcam)
Input
+H3.3-WT (dox)
+H3.3-S31A (dox)
+H3.3-S31D (dox)
+H3.3-WT (w/o dox)
+H3.3-S31A (w/o dox)
+H3.3-S31D (w/o dox)
HA

Figure 7C

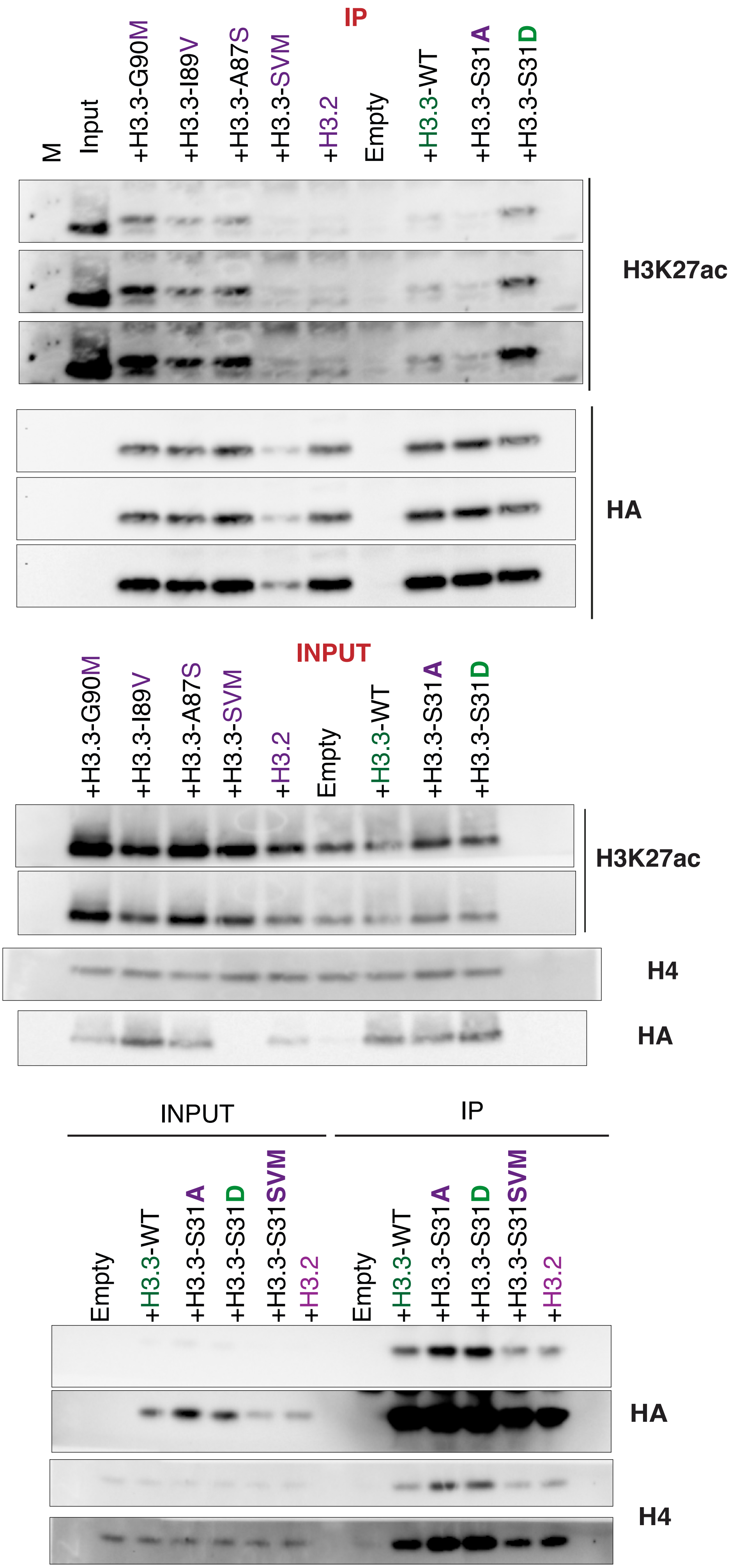
IP
M
Input
+H3.3-G90M
+H3.3-I89V
+H3.3-A87S
+H3.3-SVM
+H3.2
Empty
+H3.3-WT
+H3.3-S31A
+H3.3-S31D
H3K27ac
HA
INPUT
+H3.3-G90M
+H3.3-I89V
+H3.3-A87S
+H3.3-SVM
+H3.2
Empty
+H3.3-WT
+H3.3-S31A
+H3.3-S31D
H3K27ac
H4
HA
INPUT
IP
Empty
+H3.3-WT
+H3.3-S31A
+H3.3-S31D
+H3.3-S31SVM
+H3.2
Empty
+H3.3-WT
+H3.3-S31A
+H3.3-S31D
+H3.3-S31SVM
+H3.2
HA
H4
