# Supplementary material for: Histone variant H3.3 residue S31 is essential for Xenopus gastrulation regardless of the deposition pathway
Source: Nat Commun. 2020 Mar 9;11:1256. doi: 10.1038/s41467-020-15084-4 (PMC7062693; doi:10.1038/s41467-020-15084-4)
Supplement: Supplementary file 2 — Supplementary Information [file 41467_2020_15084_MOESM2_ESM.pdf]

Manuscript

Histone variant H3.3 residue S31 is essential for Xenopus gastrulation regardless of the deposition pathway.

Sitbon *et al.*

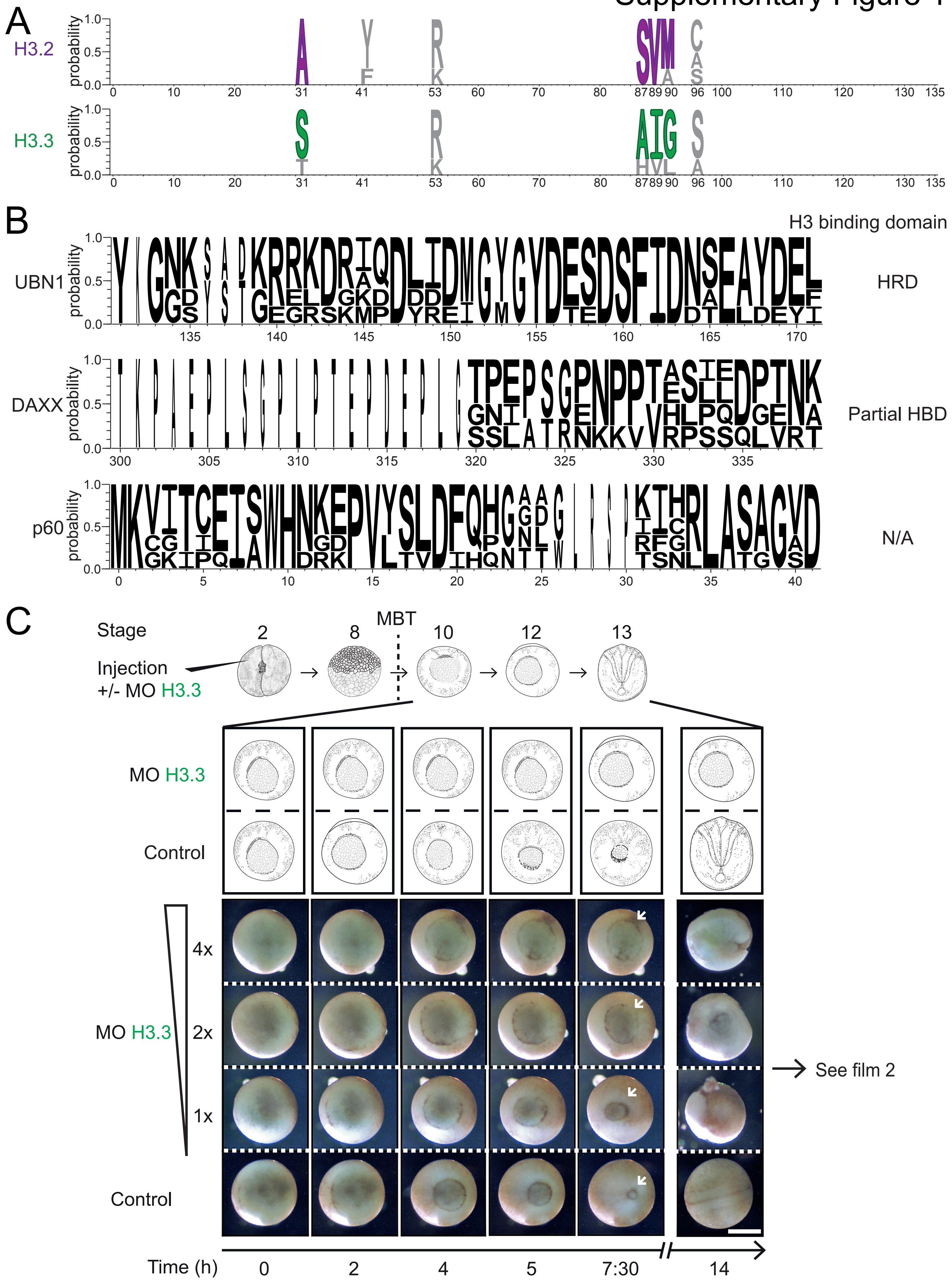
A
H3.2
H3.3
probability
B
H3 binding domain
UBN1
HRD
DAXX
Partial HBD
p60
N/A
C
Stage
MBT
Injection
+/- MO H3.3
MO H3.3
Control
4x
2x
1x
See film 2
Time (h)
0
2
4
5
7:30
14

Supplementary Figure 1: Histone chaperone sequence conservation and morpholino titration. A) Logo for H3 variant sequence differences of *Homo sapiens*, *Mus musculus*, *Drosophila melanogaster*, *Xenopus laevis*, and *Arabidopsis thaliana* after performing multiple sequence alignment using MUSCLE. Alignment displayed using WebLogo3. In addition, specific residues of each histone variant are displayed and highlighted in green and purple. B**)** Logo for UBN1, DAXX and p60 partial sequences of *Homo sapiens*, *Mus musculus*, *Drosophila melanogaster*, *Xenopus laevis* after performing multiple sequence alignment with MUSCLE. Complete histone-binding domain HRD (131-171) of UBN1 is presented, while only a part (from 300 to 339) of the DAXX histone-binding domain (HBD: 178-389) is displayed. No crystallographic data are available regarding the CAF1 complex but the overall sequence shows variation as exemplified by the first 41 residues. Final alignment are displayed using WebLogo3. **C)** Dose-dependent effects of the morpholino against endogenous H3.3. Morpholino against H3.3 is injected at the 2-cell stage with 4.6ng, 9.2ng, or 18.4ng doses. The lower concentration was previously used in [1]. White arrowheads indicate the blastopore closure. Scale bar corresponds to 500µm. See Supplementary Movie 2.

Supplementary Figure 2

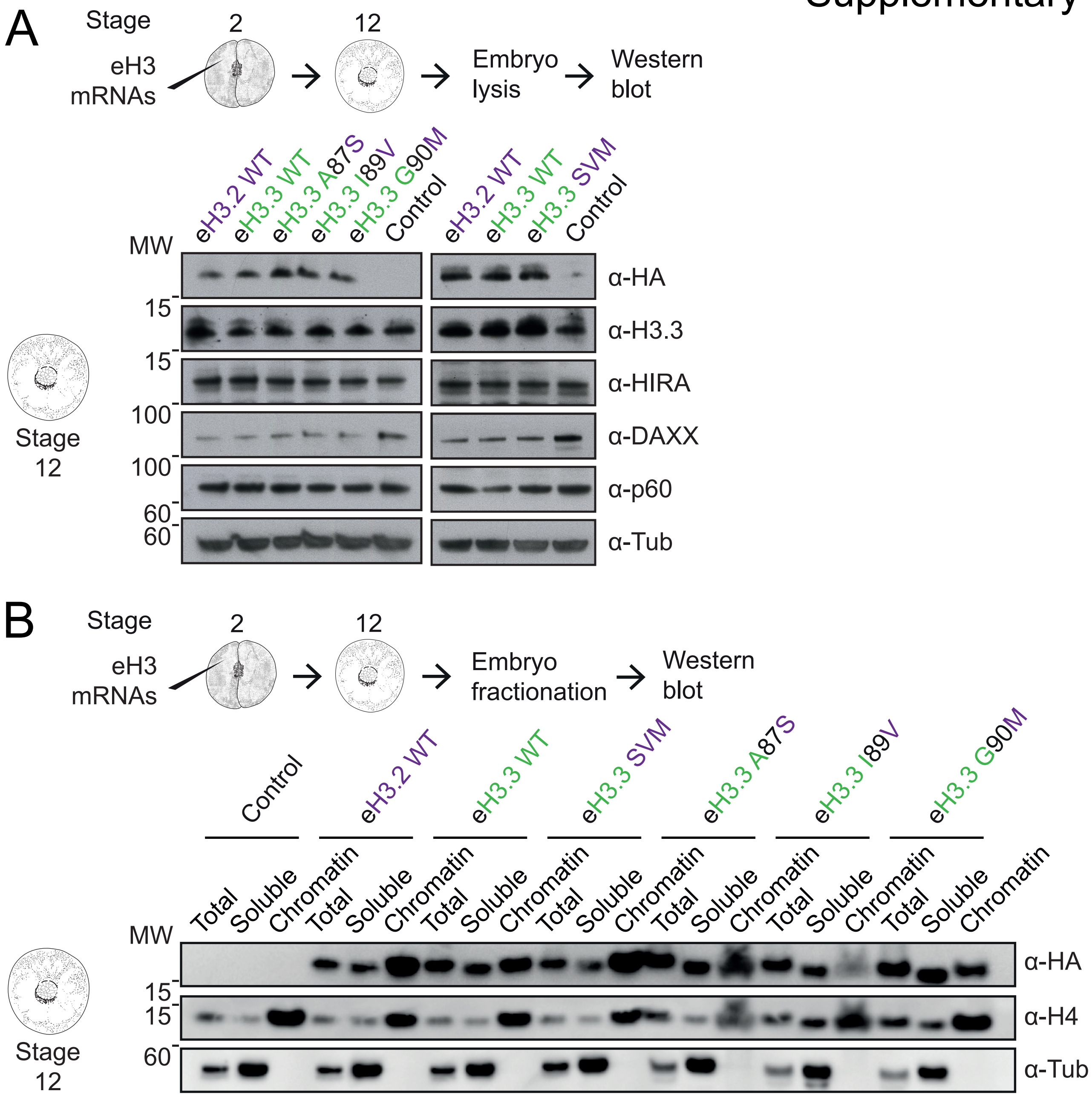

Supplementary Figure 2: eH3.3 AIG motif mutants are all expressed and incorporated in a similar fashion *in vivo*. A) Expression control of eH3.3 AIG mutants in embryos. Total embryo lysates are analyzed by western blotting with indicated antibodies. α-HA antibody is used to detect eH3.3. α-tubulin is used as an internal control. Primary antibodies indicated on the right. Molecular weight (MW) markers in kDa indicated on the left. **B)** Chromatin incorporation control of eH3.3 AIG mutants in embryos. Fractionation of stage 12 embryos injected at 2-cell stage with indicated eH3.3 mutants are shown. α-tubulin and H4 are used as fractionation controls. α-HA antibody is used to detect eH3.3. Primary antibodies indicated on the right. Molecular weight (MW) markers in kDa indicated on the left.

Supplementary Figure 3

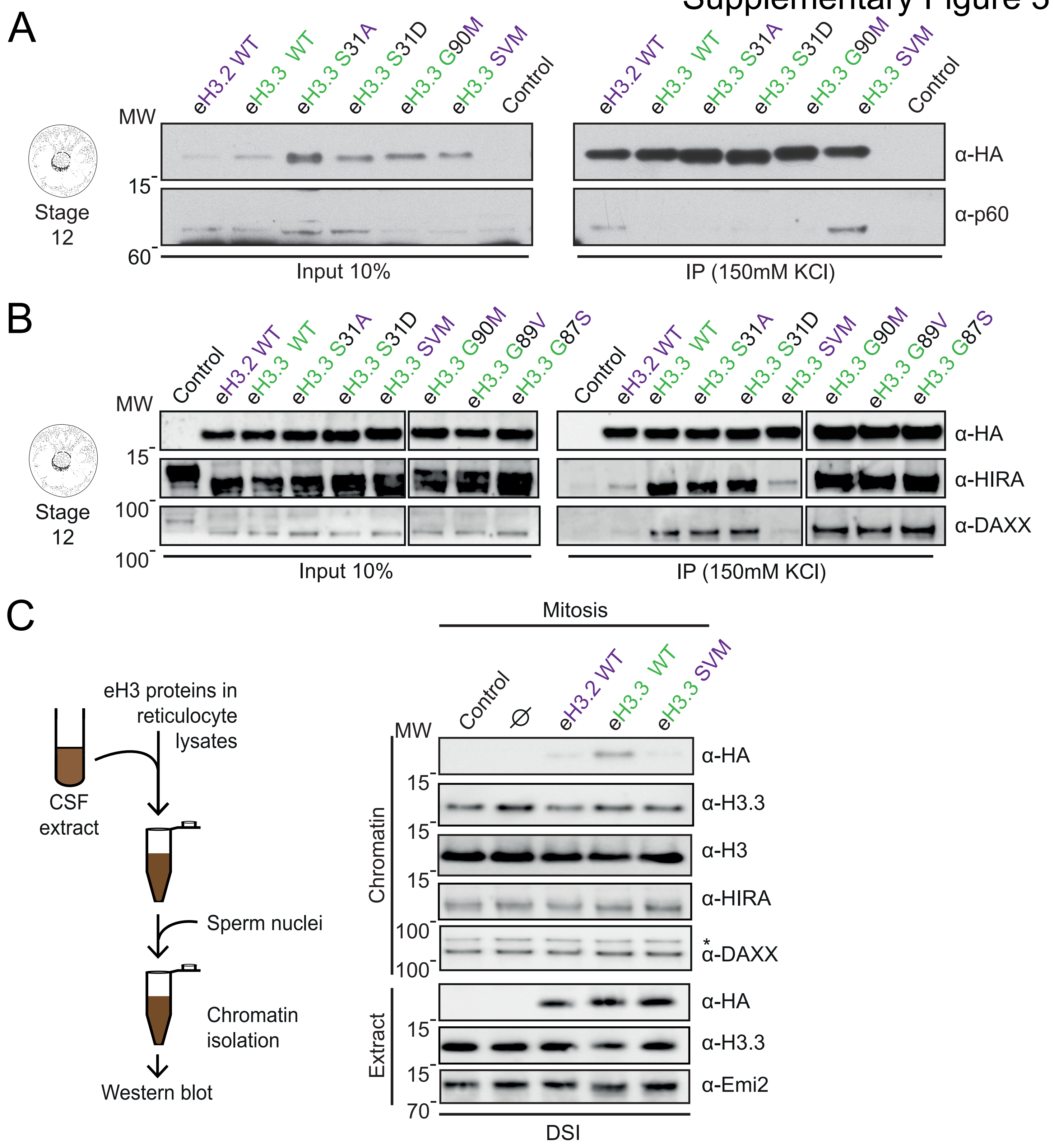

Supplementary Figure 3: Histone chaperone interactions *in vivo* and mode of incorporation of eH3.3 AIG triple mutant in mitotic extract *in vitro*. A) Immunoprecipitation of eH3 variant forms and controls from stage 12 embryos and H3.2 dedicated chaperone subunit p60. α-HA antibody is used to detect eH3.3. Primary antibodies indicated on the right. Molecular weight (MW) markers in kDa indicated on the left. **B)** Immunoprecipitation of eH3 variant forms and controls from stage 12 embryos and H3.3 dedicated chaperone HIRA and DAXX. α-HA antibody is used to detect eH3.3. Primary antibodies indicated on the right. Molecular weight (MW) markers in kDa indicated on the left. **C)** Incorporation of eH3 AIG triple mutant and controls into sperm chromatin in mitotic extract. Purified nuclei remodeled in the CSF-arrested extracts supplemented with eH3.3 mutants are analyzed by western blotting with indicated antibodies. In mitotic extract, the only available pathway is the DSI. α-HA antibodies are used to detect eH3.3. Primary antibodies indicated on the right. Molecular weight (MW) markers in kDa indicated on the left. * corresponds to a mitotic-modified form of DAXX.

Supplementary Figure 4

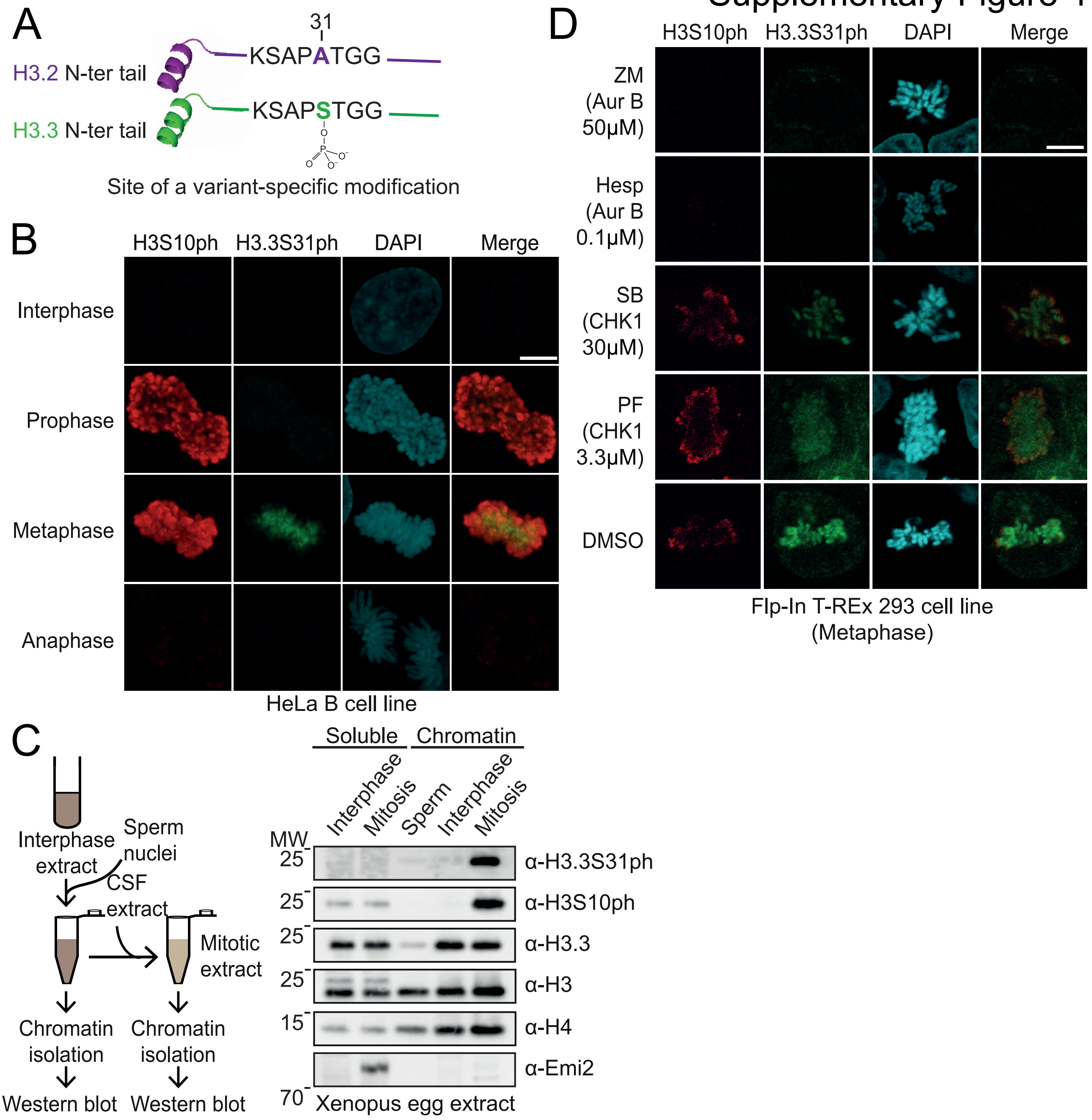

Supplementary Figure 4: H3.3S31 phosphorylation in human has similar dynamics that in *Xenopus laevis*. A) Highlights of the histone tail specific residue of H3 variants. H3.3 tail possesses a serine at position 31 that can be phosphorylated, while the alanine of H3.2 cannot. Crystal structures adapted from PDB ID codes 5B0Z [https://www.rcsb.org/structure/5B0Z] [2] and 3AV2 [https://www.rcsb.org/structure/3AV2] [3]. **B)** Representative immunofluorescence images showing 3D-distribution and timing of H3.3S31 and H3S10 phosphorylation in HeLa B cell line. Scale bar represents 10µm. **C)** Characterization of H3.3S31 phosphorylation in Xenopus cell-free extract. Non-remodeled sperm nuclei and nuclei purified after remodeling in interphase or mitotic extracts are analyzed by western blotting. Emi2 (Early mitotic inhibitor) serves as a control to confirm that extracts are in mitosis. Primary antibodies indicated on the right. Molecular weight (MW) markers in kDa indicated on the left. CSF: cytostatic factor. **D)** Effects of kinase inhibitors on H3 phosphorylation in Flp-In T-Rex 293 cell line. Asynchronous cells treated with indicated concentrations of the kinase inhibitors for 3 hours were analyzed by immunofluorescence. Mitotic cells are shown. Scale bar represents 10µm. Note: PF showed a weaker effect.

Supplementary Figure 5

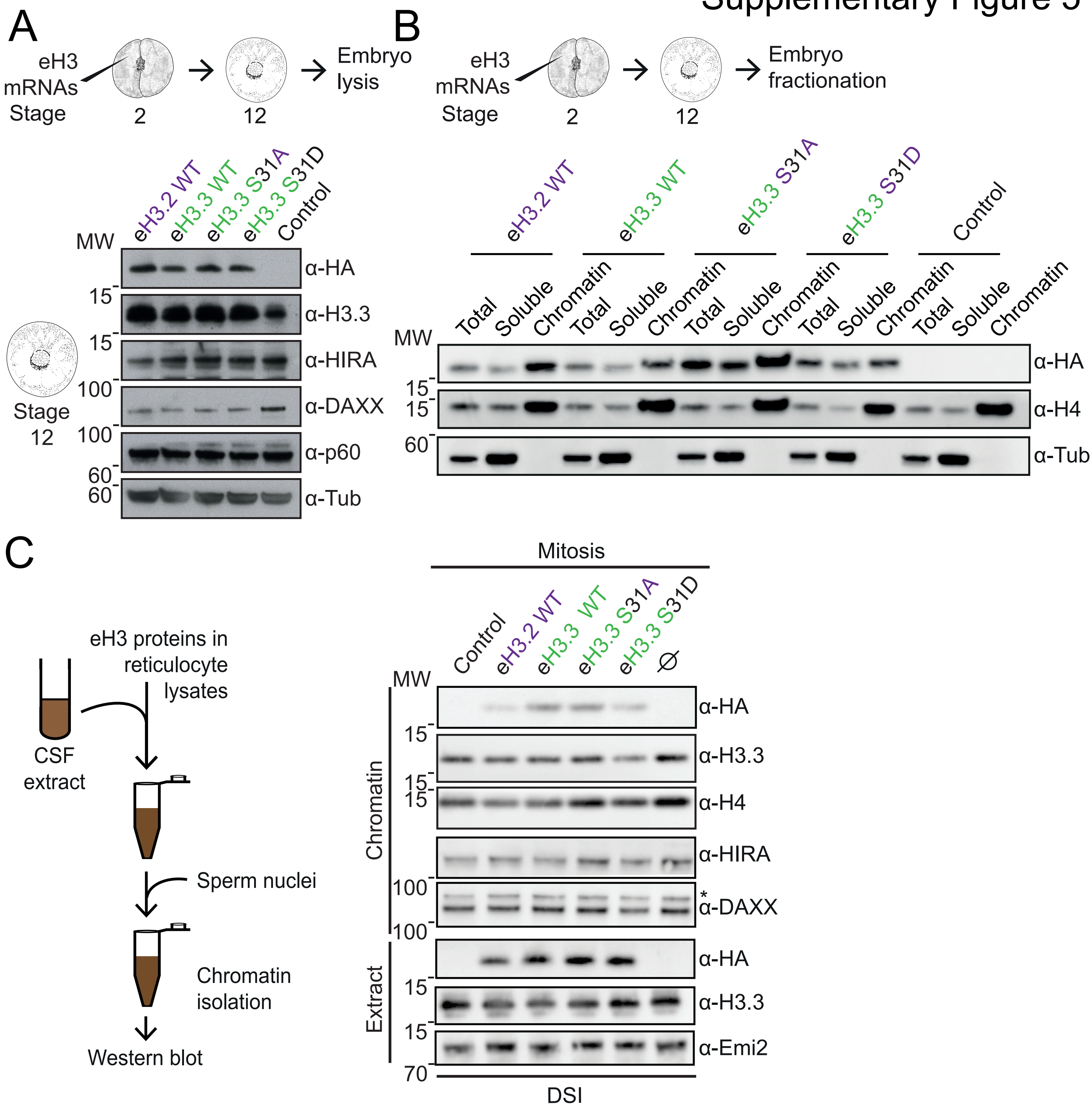

Supplementary Figure 5: Mutant forms of H3.3S31 are all expressed and incorporated in a similar fashion. A) Expression control of eH3.3S31 mutants in embryos. Total embryo lysates are analyzed by western blotting with indicated antibodies. α-HA antibody is used to detect eH3.3. α-tubulin is used as an internal control. Primary antibodies indicated on the right. Molecular weight (MW) markers in kDa indicated on the left. **B)** Chromatin incorporation control of eH3.3S31 mutants in embryos. Fractionation of stage 12 embryos injected at 2-cell stage with indicated eH3.3 mutants are shown. α-HA antibody is used to detect eH3.3. α-tubulin and H4 are used as fractionation controls. Primary antibodies indicated on the right. Molecular weight (MW) markers in kDa indicated on the left. **C)** Incorporation of eH3.3S31 mutant forms and controls into sperm chromatin in mitotic extract. Purified nuclei remodeled in the CSF-arrested extracts supplemented with eH3.3 mutants are analyzed by western blotting with indicated antibodies. In mitotic extract, the only available pathway is the DSI. α-HA antibodies are used to detect eH3.3. Primary antibodies indicated on the right. Molecular weight (MW) markers in kDa indicated on the left. * corresponds to a mitotic-modified form of DAXX.

Supplementary Figure 6

A

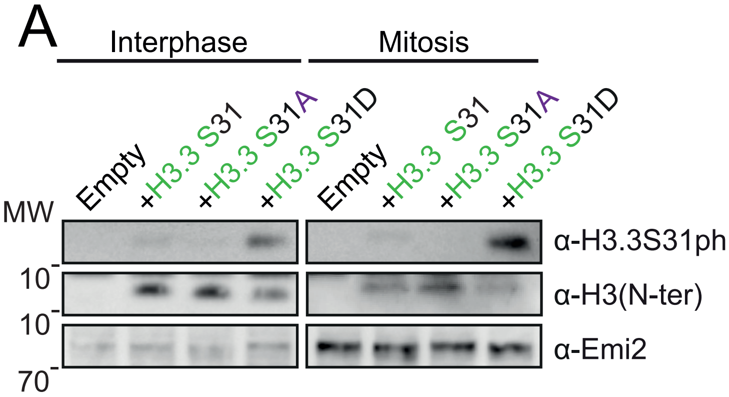

B

Gene ontology for molecular proccess

Interphase

**H3.3 S31** specific proteins (281)	p-value (10^{-3})
mitotic transition (GO:0044772)	0.000007
cell cycle G1/S (GO:0044843)	0.0038
DNA metabolic process (GO:0006259)	0.079
RNA splicing (GO:0008380)	0.085
DNA-dep replication (GO:0006261)	0.10

Metaphase

**H3.3 S31** specific proteins (93)	p-value (10^{-3})
mitochondrial transport (GO:0006839)	0.11
DNA metabo regulation (GO:0051052)	0.30
mitotic chromosome condensation (GO:0007076)	0.18
linoleic acid metabolism (GO:0043651)	0.21
glutathione biosynthetic (GO:1901687)	0.40

**H3.3 S31A** specific proteins (31)	p-value (10^{-3})
apoptotic process (GO:0042981)	5.87
neg mitotic transition (GO:1901991)	5.98
neuron apoptotic (GO:0043523)	7.51
neg reg kinase activity (GO:0033673)	7.84
interleukin-1 pathway (GO:0070498)	8.51

**H3.3 S31A** specific proteins (107)	p-value (10^{-3})
hexose biosynthetic (GO:0019319)	0.0030
tRNA aminoacylation (GO:0043039)	0.053
G1/S phase transition (GO:0044843)	0.11
mitotic transition (GO:0044772)	0.13
RNA splicing (GO:0008380)	0.21

**H3.3 S31D** specific proteins (78)	p-value (10^{-3})
cell protein assembly (GO:0043623)	0.16
bcat-TCF assembly (GO:1904837)	0.18
nucleic acid metabolism (GO:0090304)	0.20
translation preinitiation (GO:0001731)	0.26
mRNA splicing (GO:0000398)	0.33

**H3.3 S31D** specific proteins (21)	p-value (10^{-3})
nucleus organization (GO:0006997)	0.96
chromatid segregation (GO:0000070)	3.1
mRNA transport (GO:0051028)	4.8
viral life cycle (GO:0019058)	5.1
neg reg telomere capping (GO:0051028)	7.0

C

Gene ontology for molecular process of proteins common to **H3.3 S31** and **H3.3 S31A**

Interphase (66)	p-value (10^{-3})
gluconeogenesis (GO:0006094)	0.28
ESCRT disassembly (GO:1904896)	0.40
glucose metabolic (GO:0006006)	1.03
mitotic nuclear division (GO:0007088)	2.23
proteasomal ubi-indep (GO:0010499)	2.62

Metaphase (102)	p-value (10^{-3})
RNA splicing (GO:0000377)	0.013
RNA processing (GO:0006396)	0.27
macromol biosynthetic (GO:0034645)	0.29
Nter pept-methio acetyl (GO:0017196)	0.31
ATP chromatin remodel (GO:0043044)	0.39

Supplementary Figure 6: Gene ontologies analysis of the pull down proteomes with each H3 peptide. A) Peptide phosphorylation assays. Biotinylated peptides corresponding to H3 N-terminal tails are incubated in mitotic and interphase Xenopus egg extracts to check for their phosphorylation status. H3.3S31ph antibody can recognize H3.3S31D mutation. **B)** Molecular processes from gene ontology analysis for the proteins recognizing each peptide. Top 5 gene ontology was produced and ranked with the p-value score using these lists and the Enrichr tool [4]. **C)** Molecular processes from gene ontology analysis for the proteins recognizing H3.3S31 and H3.3 S31A. Top 5 gene ontology was produced with the proteins recognizing both H3.3S31 and H3.3 S31A forms and ranked with the p-value score using these lists and the Enrichr tool [4].

Supplementary Figure 7

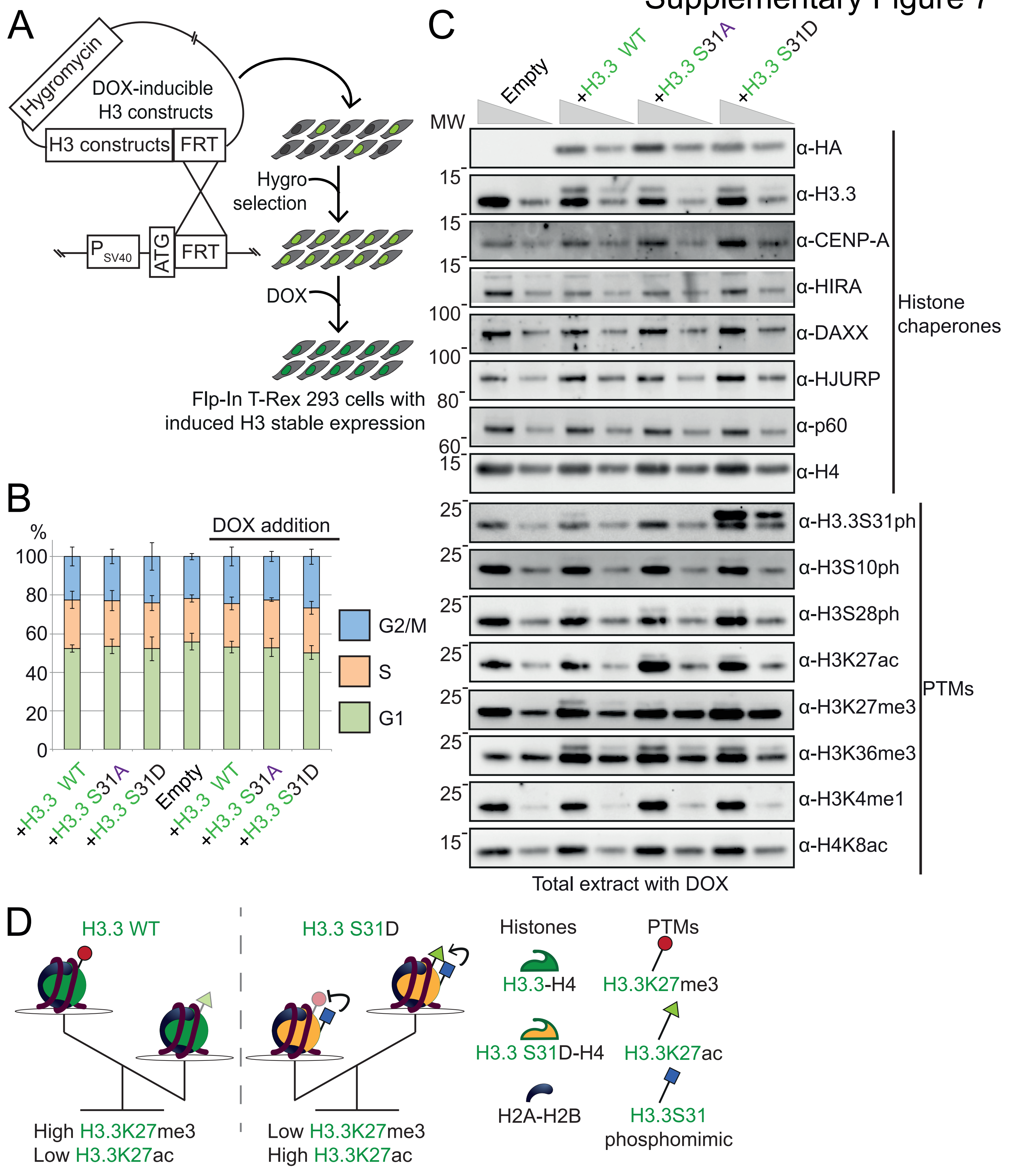

Supplementary Figure 7: Characterization of the new human Flp-In T-Rex 293 cell lines expressing inducible H3 forms. A) Construction of the Flp-In T-Rex 293 cell lines expressing inducible H3 forms. DOX-inducible H3 constructs carrying mutations for the residue at position 31 have been stably inserted into the genome of Flp-In T-Rex 293 cells and selected with Hygromycin. **B)** Cell cycle profiles for each Flp-In T-Rex 293 cell lines with or without expression of H3 constructs (DOX inducible) assessed by FACS. **C)** The levels of histone chaperones and series of PTM on H3 and H4 are analyzed by western blotting with indicated antibodies. Total extracts from $1.25x10^6$ and $6.25x10^5$ cells of DOX-induced Flp-In T-Rex 293 cell lines are loaded per lane. Primary antibodies indicated on the right. Molecular weight (MW) markers in kDa indicated on the left. **D)** Crosstalk model. H3.3 S31D mutation decreases H3.3K27me3 and instead increases H3.3K27ac in Flp-In T-Rex 293 cell lines.

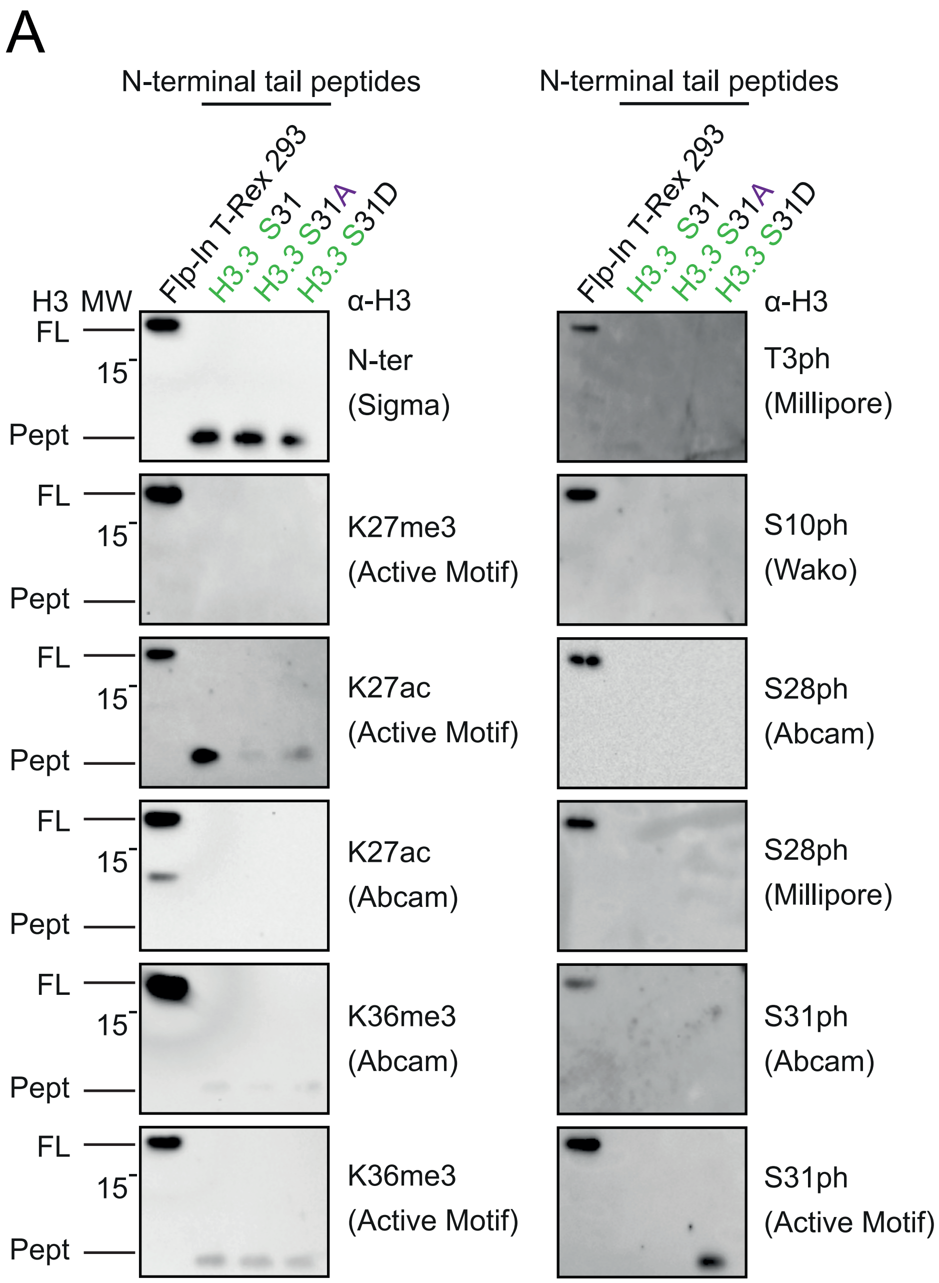
A
N-terminal tail peptides
Flp-In T-Rex 293
H3.3 S31
H3.3 S31A
H3.3 S31D
H3 MW
α-H3
FL
15
Pept
N-ter
(Sigma)
K27me3
(Active Motif)
K27ac
(Active Motif)
K27ac
(Abcam)
K36me3
(Abcam)
K36me3
(Active Motif)
T3ph
(Millipore)
S10ph
(Wako)
S28ph
(Abcam)
S28ph
(Millipore)
S31ph
(Abcam)
S31ph
(Active Motif)

Supplementary Figure 8: H3 PTM antibody crosstalk with unmodified H3 peptides. 30pmol of each peptide, or $8.3x10^3$ cells as positive control, have been loaded for western blotting analysis using antibody targeting H3 PTMs. We only excluded H3K27ac from Active Motif since it recognized unmodified H3.3 N-terminal peptides. FL: full length H3, Pept: H3 peptides.

Supplementary References

1 Szenker, E., Lacoste, N. & Almouzni, G. A developmental requirement for HIRA-dependent H3.3 deposition revealed at gastrulation in Xenopus. *Cell Rep* **1**, 730-740, doi:10.1016/j.celrep.2012.05.006 (2012).

2 Suzuki, Y., Horikoshi, N., Kato, D. & Kurumizaka, H. Crystal structure of the nucleosome containing histone H3 with crotonylated lysine 122. *Biochem Biophys Res Commun* **469**, 483-489, doi:10.1016/j.bbrc.2015.12.041 (2016).

3 Tachiwana, H. *et al.* Structures of human nucleosomes containing major histone H3 variants. *Acta Crystallogr D Biol Crystallogr* **67**, 578-583, doi:10.1107/S0907444911014818 (2011).

4 Chen, E. Y. *et al.* Enrichr: interactive and collaborative HTML5 gene list enrichment analysis tool. *BMC Bioinformatics* **14**, 128, doi:10.1186/1471-2105-14-128 (2013).
